# Genome Sequence of an Alphabaculovirus Isolated from the Oak Looper, *Lambdina fiscellaria, *Contains a Putative 2-Kilobase-Pair Transposable Element Encoding a Transposase and a FLYWCH Domain-Containing Protein

**DOI:** 10.1128/genomeA.00186-15

**Published:** 2015-05-28

**Authors:** George F. Rohrmann, Martin A. Erlandson, David A. Theilmann

**Affiliations:** a Department of Microbiology, Oregon State University, Corvallis, Oregon, USA; b Saskatoon Research Centre, Agriculture and Agri-Food Canada, Saskatoon, Saskatchewan, Canada; c Pacific Agri-Food Research Centre, Agriculture and Agri-Food Canada, Summerland, British Columbia, Canada

## Abstract

The genome sequence of an alphabaculovirus isolated from *Lambdina fiscellaria* indicated that it is a novel member of a group II lineage. A putative transposable element was identified that contained two genes, including a transposase ortholog. These genes were most closely related to genes of the pea aphid*, Acyrthosiphon pisum.*

## GENOME ANNOUNCEMENT

A nucleopolyhedrovirus (NPV) associated with oak looper (*Lambdina fiscellaria*) (Lepidoptera: Geometridae) (LafiNPV, also known as isolate GR15) larvae was part of an archival collection of previously characterized infected insects (1). A partial *lef8* gene sequence indicated it was a member of a group II lineage of alphabaculoviruses and was significantly different from other members of that lineage (1). In order to further characterize this virus, the genome was sequenced and determined to be 157,989 bp, with a G+C content of about 44%, and it contains 135 predicted open reading frames of ≥25 amino acids. Orthologs of all 37 baculovirus core genes were identified.

The relatedness of baculovirus genomes is normally determined by combining PIF2 and LEF8 amino acid sequences. The sequence of both these genes from LafiNPV showed the closest relatedness to sequences from the group II lineage that includes *Orgyia leucostigma* NPV, *Euproctis pseudoconspersa* NPV, and *Buzura suppressaria* NPV, with amino acid sequence identities of 74 to 76% (PIF2) and 64% (LEF8), suggesting that it represents a new alphabaculovirus species. In addition, the polyhedrin sequence was almost identical to that reported in other LafiNPVs (2), suggesting a close relationship with Eastern and Western hemlock looper NPV isolates.

P26 is a protein encoded by the genomes of all group I and most group II alphabaculoviruses, but is not present in other baculovirus genera, although orthologs have been found in some poxviruses. It does not appear to be an essential gene, and its function is not known (3). Several group II baculovirus genomes contain two complete *p26* genes. However, in the LafiNPV genome sequence, one of the two genes was found to be separated by the insertion of a transposable element of 2 kb at amino acid 134, resulting in N- and C-terminal *p26*-related open reading frames (ORFs) of 134 (Lafi117) and 162 (Lafi114) amino acids, respectively. The transposable element contained a transposase ortholog most closely related (BLASTp expected value, 8e-37) to an ORF from the pea aphid, *Acyrthosiphon pisum*, and an adjacent ORF most closely related to an ORF (BLASTp expected value, 1e-04) also from *A. pisum*. Analysis of the second ORF using HHpred (4) resulted in a 98% probability (expected value, 7.5E-07) of structural similarity to the fifth FLYWCH domain of FLYWCH-type zinc finger-containing protein 1 in humans (5). Although FLYWCH is a C2H2-type zinc finger involved in protein-protein interactions (6), the LafiNPV FLYWCH domain is a C2HC-type zinc finger. The two ORFs were flanked by inverted repeats of 118 nucleotides (nt), and 12 nt at one end of each repeat is identical to the *p26* sequence where the insert occurred. The relatively distant relationship of the transposable element ORFs to those from *A. pisum* suggests that the insertion event in the LafiNPV genome occurred in the distant past, or it has moved through other hosts over a significant period of time before insertion in the LafiNPV genome.

### Nucleotide sequence accession number.

The genome of LafiNPV has been deposited in GenBank under the accession no. KP752043.
